# Groundwater helium anomaly reflects strain change during the 2016 Kumamoto earthquake in Southwest Japan

**DOI:** 10.1038/srep37939

**Published:** 2016-11-29

**Authors:** Yuji Sano, Naoto Takahata, Takanori Kagoshima, Tomo Shibata, Tetsuji Onoue, Dapeng Zhao

**Affiliations:** 1 Atmosphere and Ocean Research Institute, The University of Tokyo, Kashiwa, Chiba 277-8564, Japan; 2 Institute for Geothermal Sciences, Kyoto University, Beppu, Oita 874-0903, Japan; 3 Department of Earth and Environmental Sciences, Kumamoto University, Kurokami, Kumamoto 860-8555, Japan; 4 Department of Geophysics, Tohoku University, Sendai, Miyagi 980-8578, Japan

## Abstract

Geochemical monitoring of groundwater and soil gas emission pointed out precursor and/or coseismic anomalies of noble gases associated with earthquakes, but there was lack of plausible physico-chemical basis. A laboratory experiment of rock fracturing and noble gas emission was conducted, but there is no quantitative connection between the laboratory results and observation in field. We report here deep groundwater helium anomalies related to the 2016 Kumamoto earthquake, which is an inland crustal earthquake with a strike-slip fault and a shallow hypocenter (10 km depth) close to highly populated areas in Southwest Japan. The observed helium isotope changes, soon after the earthquake, are quantitatively coupled with volumetric strain changes estimated from a fault model, which can be explained by experimental studies of helium degassing during compressional loading of rock samples. Groundwater helium is considered as an effective strain gauge. This suggests the first quantitative linkage between geochemical and seismological observations and may open the possibility to develop a new monitoring system to detect a possible strain change prior to a hazardous earthquake in regions where conventional borehole strain meter is not available.

Volatile element degassing from the solid Earth has been a major subject of geochemistry because it may provide a constraint on the evolution of atmosphere and ocean^1^ together with mechanism of climate change and environmental consequence of volcanism^2^,^3^. Even though magmatic and hydrothermal fluxes are spectacular and have a high profile^4^,^5^, gas emanations related to seismic and fault activities are not negligibly small and may contribute atmospheric mass balance^6^,^7^,^8^. Geochemical monitoring of groundwater and soil gas emission has been conducted for earthquake prediction in USA, Japan, China and Italy since 1970s^9^. Precursor and/or coseismic anomalies of noble gases such as groundwater radon and helium/argon ratios were well documented^10^. Several years prior to an earthquake in Tashkent, radon contents in deep wells increased to about double normal values. Soon after the earthquake, they returned to normal^11^. It was the first report of radon anomaly and stimulated many following researchers. During the 1995 Kobe earthquake, continuous radon-monitoring was made at a site located about 30 km northeast of the epicenter^12^. The radon concentration at the end of 1993 was stable at 20 Bq per liter. Radon started to increase gradually from October 1994 to the end of November 1994, reaching about 60 Bq per liter, which was three times that of the normal level. A sudden increase was seen on January 7, 1995 and then decreased on January 10, a week before the Kobe earthquake. Since then the radon content returned to the November 1993 level. It was the most significant precursor of a large earthquake. In addition, conspicuous changes in He/Ar ratios were observed at a fumarole of Mt Ontake and a mineral spring in the Byakko just before the occurrence of an inland earthquake (M 6.8) in central Japan in September 1984; the fumarole and the spring are located 9 and 50 kilometers, respectively, from the epicenter^13^. The anomaly was probably resulted from deep-seated fluids being squeezed to the surface by the tectonic stress that caused the earthquake. However there was lack of a plausible physico-chemical basis to explain these phenomena^14^. A laboratory experiment of rock fracturing and helium and radon emission was conducted^15^,^16^, but there is no quantitative relationship between the laboratory results and field observation. It is necessary to connect gas geochemistry, model of strain change and rock fracturing experiment in a comprehensive study.

The 2016 Kumamoto earthquake (M 7.3 in the Japan Meteorological Agency scale)^17^ occurred in Kumamoto City on Kyushu Island, Southwest Japan on April 2016, causing 50 fatalities, over 1800 injured and serious damage to local infrastructures. It was an inland crustal earthquake with a strike-slip fault and a shallow hypocenter (10 km depth) close to highly populated areas (see Methods). In order to study geochemical state of the Kumamoto area, we collected groundwater samples. There are noble gas data collected and measured in the same areas in August 2010^18^. So it is possible to compare directly the helium isotopes before and soon after the Kumamoto earthquake. Based on the data, we discuss the connection between emanation of crustal helium, estimated volumetric strain change, and a quantitative comparison with a rock fracturing experiment.

## Results

### Helium isotopes of groundwater samples

**Figure 1:**
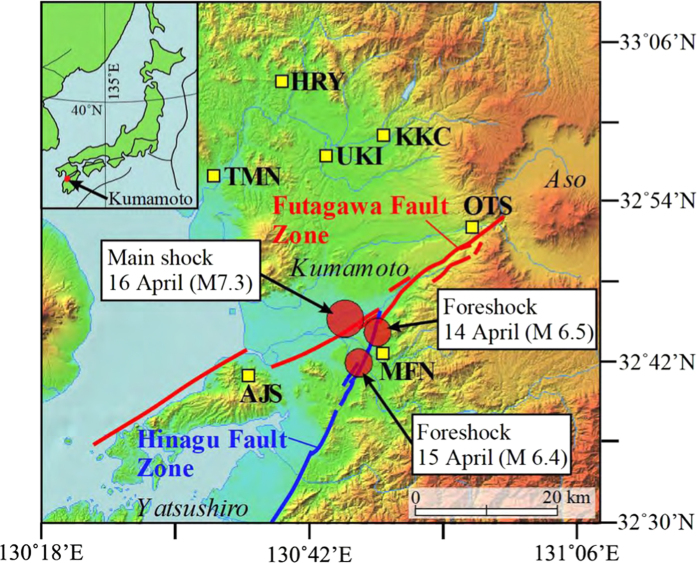
Sampling sites of deep groundwater in the Kumamoto region in Southwest Japan together with epicenters of two foreshocks (M 6.5 and M 6.4) and the main shock (M 7.3) of the 2016 Kumamoto earthquake, as well as the Futagawa-Hinagu fault zones. The base map is modified from the Digital Japan Portal Web Site, Geospatial Information Authority of Japan (http://maps.gsi.go.jp).

**Figure 2:**
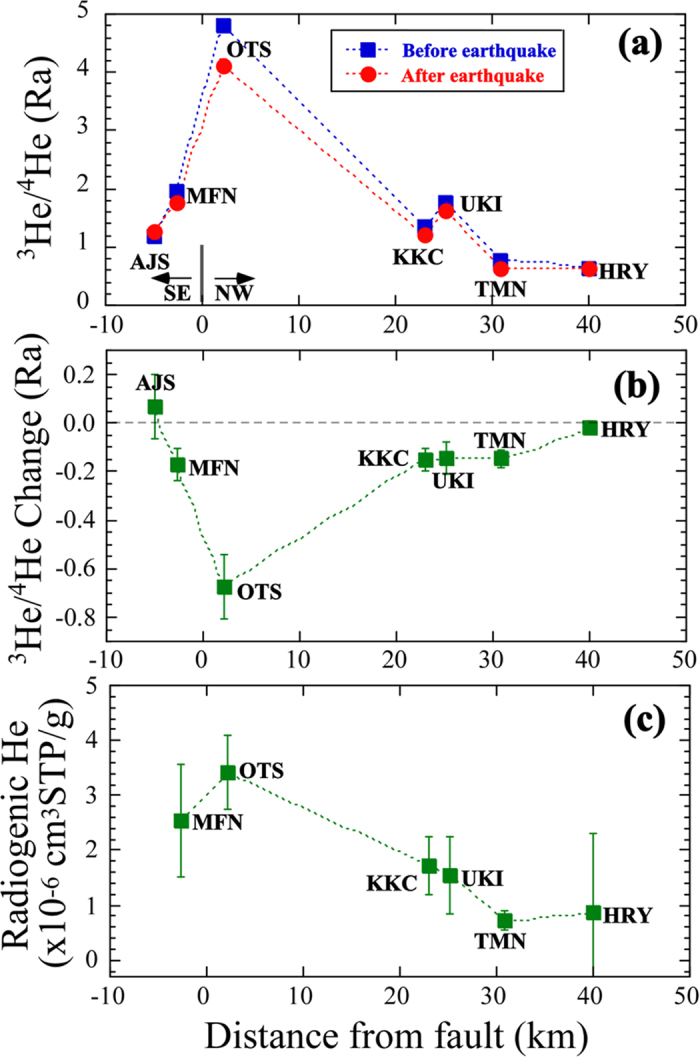
Relationship between the distance from the fault to the sampling site and (**a**) ^3^He/^4^He ratios of deep groundwater before and after the M 7.3 earthquake, (**b**) that between the distance and the ^3^He/^4^He change, and (**c**) that between the distance and the radiogenic helium abundance released after the M 7.3 earthquake. Errors are two sigma values.

Seven deep groundwater samples (280–1300 m) were collected in the Futagawa-Hinagu fault zones during April 28 to 29, 2016 (Fig. 1), eleven days after the Kumamoto main shock. Helium and neon abundances and helium isotopes were measured by conventional mass spectrometers (Methods). The ^3^He/^4^He ratios of samples derived from the upper mantle show a high value of 8 Ra (Ra is the ^3^He/^4^He ratio of 1.382 × 10^−6^)^19^, whereas those of crustal fluids are characterized by a radiogenic ratio of ~0.02 Ra^20^. Observed ^3^He/^4^He ratios of our samples vary from 0.623 Ra to 4.12 Ra (STable 1) and show that groundwater in the Futagawa-Hinagu fault zones contains mantle helium contributions of ~7% to ~50%. The highest ratio was found in Otsu hot spring, close to the northeastern end of the Futagawa fault where the surface rapture was found (Fig. 1). An elevated ^3^He/^4^He ratio was also observed in Mifune hot spring, close to the epicenter of a major foreshock (M 6.4) on April 15. However, the ratios are lower than the air value at distant sites from the faults, such as Tamana and Hirayama hot springs. Figure 2a shows a relationship between the approximate distances from the Futagawa-Hinagu fault to the sampling sites and the observed ^3^He/^4^He ratios of our samples together with those measured six years before the 2016 Kumamoto earthquake^18^. The overall variations are consistent before and soon after the earthquake, and there is a modest trend of decreasing ratios with distance away from the Futagawa-Hinagu fault. The same trend was first recognized in the San Andreas fault system in California^21^ and confirmed by later studies in the North Anatolian fault zone in Turkey^22^ and the Karaforam fault in Tibet^23^. Note that they are all long strike-slip faults, and a general explanation of the location-dependent variation is due to influx of mantle fluids as follows: helium with a high ^3^He/^4^He ratio exsolving from partially-melted zones in the upper mantle becomes focused at the bottom of a major crustal fault, and subsequently traverses the entire crust via a permeable fault plane. During the uprising of the fluids, they may be diluted by radiogenic helium with a low ratio. At the distant sites from the fault, the crustal dilution may be profound. This is the case for our samples in the Kumamoto region.

### Temporal variations of helium isotopes

The location-dependent variations of ^3^He/^4^He ratios are similar before and after the 2016 Kumamoto earthquake as stated above (Fig. 2a). However, their temporal changes are different at particular sites. At Otsu hot spring, 2.2 km northwest of the Futagawa-Hinagu fault, the ^3^He/^4^He ratio decreased from 4.795 ± 0.088 Ra (all errors are 2σ hereafter) in August 2010 to 4.120 ± 0.100 Ra in April 2016. Another lowering of the ratio from 1.959 ± 0.056 Ra to 1.780 ± 0.040 Ra was found at Mifune hot spring, 2.7 km east of the Hinagu fault. Although Mifune is located closest to the main shock epicenter, its helium isotopes decrease is smaller than that at Otsu. This is partly due to the difference of original ^3^He/^4^He ratios. If we assume that the same amount of crustal helium, 2.6 × 10^−6^ cm^3^ STP/g, was added into the Otsu and Mifune hot springs, decrease of the ^3^He/^4^He ratio becomes 0.50 Ra at Otsu, larger than 0.17 Ra at Mifune. There is no statistically valuable change of the ratio in Hirayama hot spring, 40 km away. Figure 2b shows a relationship between the distance from the fault and ^3^He/^4^He changes before and soon after the earthquake. The temporal changes are relatively large in the area close to the fault and they are negligibly small at the both ends, Hirayama and Ajisai hot springs. It is, perhaps, a mirror image of the location-dependent variations (Fig. 2a), suggesting that the decrease of ^3^He/^4^He ratio occurred significantly in the place where the original mantle contribution was large.

### Helium isotopes of rock samples

We have also measured helium abundance and isotopic ratios of six rock samples, which are derived from outcrops in the Kumamoto region and geologically considered to be an aquifer rock system of deep groundwater such as those at Mifune and Otsu (STable 2). The average He abundance of (1.9 ± 0.9) × 10^−6^ cm^3^ STP/g is nearly the same as in the Kobe aquifer rocks^7^ (2.1 ± 1.3) × 10^−6^ cm^3^ STP/g and is well within the variation of helium contents, (6.1 ± 4.3) × 10^−6^ cm^3^ STP/g, in crustal rocks adopted by the rock fracturing experiment^15^. On the other hand, the ^3^He/^4^He ratios of the Kumamoto samples, vary significantly from <0.01 Ra to 0.56 Ra, even though their average with a weighted error of 0.09 ± 0.02 Ra (MSWD = 0.90) is apparently higher than that of the Kobe samples, 0.02 ± 0.01 Ra^7^.

## Discussion

The temporal variations of ^3^He/^4^He ratios correlated with the distance from the fault (Fig. 2b) support the hypothesis that decrease of the ratio was strongly affected and perhaps invoked by the 2016 Kumamoto earthquake. Helium is an inert gas and its isotopic composition is not subject to biological-chemical reactions, but due to diffusion, partition, and radioactive decay^20^. It is necessary to explain the temporal variations by physical means. First, we consider absolute concentrations of ^4^He and ^20^Ne in groundwater samples in the region. The ^4^He contents decreased at a few hot springs after the earthquake, whereas there are no meaningful changes at the other sites. There is no systematic relation with the distance and variation (SFig. 1a). The ^20^Ne contents also showed non-systematic change (SFig. 1b). It is difficult to explain the temporal variations of the noble gas abundances related to the M 7.3 earthquake. Second, we consider the ^4^He/^20^Ne ratios together with the ^3^He/^4^He ratios. Generally, a three-component mixing model was taken into account in subduction zones based on these ratios^24^. Their end members are air saturated water (ASW) with ^3^He/^4^He = 1 Ra, ^4^He/^20^Ne = 0.268; upper mantle with ^3^He/^4^He = 8 Ra, ^4^He/^20^Ne = 1000; and radiogenic with ^3^He/^4^He = 0.02 Ra, ^4^He/^20^Ne = 1000, respectively. Our samples together with those before the M 7.3 earthquake are located well within the mixing region of the three components in ^4^He/^20^Ne–^3^He/^4^He diagram (SFig. 2). The ^3^He/^4^He ratios at most sites decreased after the earthquake (Fig. 2b). At the same time, the ^4^He/^20^Ne ratios also decreased. The variations cannot be attributed to simple enhancement of air contribution, because the sample with a ^3^He/^4^He ratio lower than the air value (e.g. Tamana hot spring) also shows a decrease in the ^4^He/^20^Ne ratio. In addition, changes of a few samples (Tamana and Ueki hot springs) are not approaching into the ASW value (SFig. 2). This suggests that the temporal variation was derived from the addition of radiogenic helium together with the air component. The latter component may be attributable to the mixing of shallow and air saturated groundwater probably induced by the M 7.3 earthquake.

It is possible to correct the ^3^He/^4^He ratio for diminishing air contribution based on the observed ^4^He/^20^Ne ratio^25^. If the ^4^He/^20^Ne ratio is close to its air value, the correction could be significantly erroneous^26^. Therefore we did not take into account the Ajisai samples with low ^4^He/^20^Ne ratios of less than 0.5 in following discussion, because more than 50% of their helium is derived from air. Then we can compare the corrected ^3^He/^4^He ratios before and soon after the M 7.3 earthquake in order to estimate the increase of radiogenic helium (STable 3). Again it is possible to estimate the amount of additional crustal helium using the change of the corrected ^3^He/^4^He ratio and original abundance of helium in groundwater sample in 2010^18^ under the assumption that ^3^He fluxes into hot springs are constant and helium released from aquifer rocks has a ^3^He/^4^He ratio of 0.09 Ra (STable 2). The larger addition of helium is found at Otsu and Mifune hot springs, while Tamana and Hirayama show a relatively small value. Figure 2c indicates a relationship between distance from the fault and additional helium abundance in groundwater. There is a striking trend of emanation with the distance, suggesting that the additional helium may be attributable to the M 7.3 earthquake.

We should discuss the mechanism of crustal helium degassing due to the earthquake. At the time of the 1995 Kobe earthquake (M 7.2), similar degassing of helium was observed in shallow groundwater at Nishinomiya city, 30 km northeast of the epicenter^7^. The degassing was attributed to release of radiogenic helium accumulated in country rock as a result of micro-fracturing during the earthquake. It is possible to calculate the amount of helium released from aquifer rock into deep groundwater. Porosity of deep aquifer (ϕ) was reported to be approximately 10% in the Kumamoto region^27^. Assuming the density of country rock (ρ) to be 2.8 g/cm^3^ and taking the porosity into account, additional helium in groundwater would be converted into absolute amount of helium released from the aquifer rock as follows: [He]_water_ × ϕ/{(1 − ϕ)ρ}. This value is approximately 1.1–1.4 × 10^−7^ cm^3^ STP/g at Otsu and Mifune hot springs close to the fault, two orders of magnitude greater than that of 1.4 × 10^−9^ cm^3^ STP/g during the Kobe earthquake^7^. The discrepancy may be due to the difference of aquifer depth (3–20 m in Kobe and 300–1000 m in Kumamoto) and distance from the earthquake fault (10 km in Kobe and 2 km in Kumamoto), or volumetric strain change. On the other hand, additional helium of Hirayama hot spring at the end is consistent with zero within the experimental error range (STable 3). These geographical variations may be related to the strain change of the source zone and/or fracturing of aquifer rock affected by the M 7.3 earthquake.

**Figure 3:**
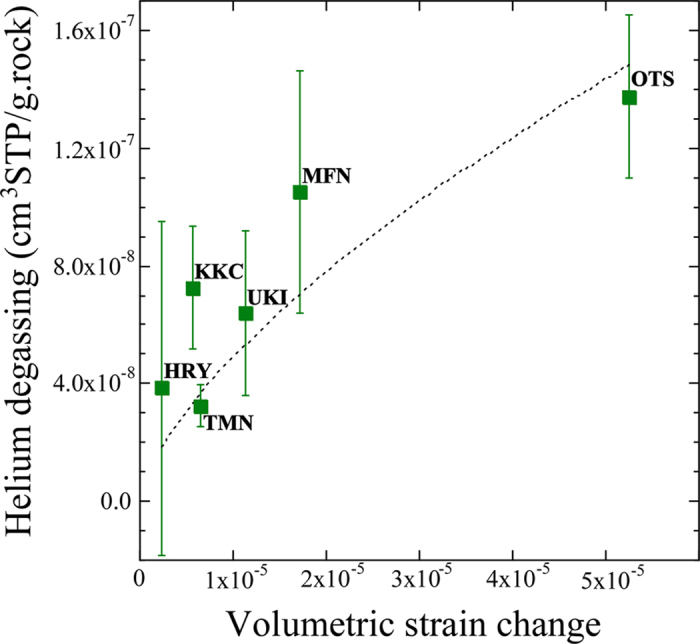
Relationship between the calculated volumetric strain change and abundance of released radiogenic helium at the sampling sites. Errors are two sigma values. The dotted curve shows a least-squares fitting of equation (1).

Coseismic volumetric strain changes at every sites are calculated using a fault model (Methods) and geodetic data reported by Geospatial Information Authority of Japan^28^,^29^. There are three data sets for each fault, A1, A2 and B (STable 3). Strain changes are variable with time and space. For example, it is −2.04 × 10^−6^ for A1 fault, 1.70 × 10^−6^ for A2, and −1.95 × 10^−6^ for B in Kikuchi site, respectively. We calculate the summation of absolute values because either positive strain change (extension) or negative (compression) may contribute rock fracturing and degassing. Although Mifune is located closer to the main shock epicenter than Otsu, its total strain change is smaller than the Otsu according to the calculation results (STable 3). This may explain the smaller decrease of the ^3^He/^4^He ratio at Mifune than that at Otsu. There is an apparent positive correlation between the total strain changes and amounts of released helium at each site (Fig. 3). This is the first data set of quantitative linkage between seismological and geochemical observations, which would be explained by a physical mechanism such as release by rock fracturing. Assuming that the amount of helium (υ) released from aquifer rock is proportional to the newly exposed surface area^15^, we may write the relation between the volumetric stain change (ΔV/V) and amount of released helium (υ) as:


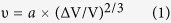


where *a* is a constant and equal to k_2_ × υ_0_ (k_2_: constant obtained by rock fracture experiment in laboratory, υ_0_: initial amount of helium of the aquifer rock). Least-squares fitting of data in the relationship (a dotted curve in Fig. 3) provides the value of unknown parameter as follows: *a* = (1.06 ± 0.13) × 10^−4^, where R = 0.852 and MSWD = 0.84, suggesting that the fitting is statistically valid. In the fault model and/or actual field observation during the earthquake, volumetric strain change may take a positive (extension) or negative (compression) value. On the other hand, it is difficult to conduct a rock fracturing experiment by extension mode in laboratory. Then we take into account the summation of absolute values of calculated strain changes for ΔV/V. Based on the estimated values *a* = 1.06 × 10^−4^ and k_2_ = 25 ± 15 from the fracturing experiment^15^, the initial amount of helium of the aquifer rock is obtained to be (4.2 ± 2.6) × 10^−6^ cm^3^ STP/g. The estimated helium abundance is generally consistent with the observed value of (1.9 ± 0.9) × 10^−6^ cm^3^ STP/g in the hypothetical Kumamoto aquifer rocks within the experimental error range (STable 2) and it is comparable to the average of helium contents, (6.1 ± 4.3) × 10^−6^ cm^3^ STP/g, in crustal rocks adopted by the rock fracturing experiment^15^.

## Conclusions

We found a quantitative relationship between deep groundwater helium anomaly and volumetric strain change during the 2016 Kumamoto earthquake in Southwest Japan. The correlation is plausibly explained by the generation of new surface area of rock by dilatancy in the laboratory experiment, suggesting that the groundwater helium with its abundance and isotopic composition may act as effective volumetric strain gauge. Even though this phenomena should be verified in other earthquake areas, it may lead to development of a new geochemical monitoring system to detect possible strain changes prior to a hazardous earthquake in regions where adequate geophysical observation is not available.

## Methods

### The 2016 Kumamoto earthquake

The Kumamoto earthquake sequence began with a strong foreshock (M 6.5) at local time 21:26 on 14 April 2016 with a focal mechanism of strike-slip faulting at a focal depth of ~11 km. Another big foreshock (M 6.4) occurred at 00:03 on 15 April, 7 km southwest of the first one (Fig. 1). Then the main shock (M 7.3) took place at 01:25 on 16 April in the vicinity of the two foreshocks. Its focal mechanism is also strike-slip faulting with northwest-southeast tension, and its focal depth is ~10 km. None of its nodal planes are consistent with the surface traces of the known Futagawa-Hinagu fault zones, which are the most prominent active faults in the Kyushu region. The aftershocks are generally located along the Futagawa-Hinagu fault zones and distributed at depths of 3–17 km dipping toward the northwest^30^. Surface fault ruptures associated with the main shock were clearly present along the Futagawa fault with a right-lateral strike-slip offset of up to 2 m^31^. Another offset was observed along the northernmost Hinagu fault. The main shock caused extensive damage to the infrastructure along the Futagawa fault with a peak ground acceleration of 1580 cm/s^2^, killing at least 50 inhabitants, and over 1,800 people were injured by house collapses and subsequent land slides^32^. About 8,300 houses were completely destroyed, some 2,600 half-damaged, and roughly 125,800 partially damaged by severe ground shaking^33^.

### Sampling and analysis

At the sampling sites, groundwater was drawn continuously from deep well by electric submersible pump for at least a couple of hours. Then a 50 cm^3^ lead glass container with vacuum valves at both ends were connected with the faucet using a tick wall plastic tube. After approximately 10 minute of flushing of groundwater through the entire system, both valves were closed. Sample exposure to ambient air was significantly minimized during the process. In the laboratory, dissolved gases were extracted by a head space method in ultra-high vacuum. A portion of gases was introduced into all metal purification and separation vacuum line, and helium and neon were refined. The ^4^He/^20^Ne ratios together with ^4^He and ^20^Ne abundances were measured by a quadrupole mass spectrometer, while the ^3^He/^4^He ratios were determined by a VG5400 noble gas mass spectrometer at Atmosphere and Ocean Research Institute, the University of Tokyo^34^.

### Volumetric strain change

Coseismic volumetric strain changes at every sampling sites are calculated for a fault model^35^ using geodetic data reported by Geospatial Information Authority of Japan (GSI), which are estimated from ground displacements detected by interferometric synthetic aperture radar (InSAR) of satellite and global navigation satellite system (GNSS). The data were inverted for three rectangular faults with uniform slips in an elastic half-space, A1, A2 and B, related to the 2016 Kumamoto earthquake sequence with two major foreshocks (M 6.5 and M 6.4) and the main shock (M 7.3) (SFig. 3). Then total strain changes at the sampling sites were estimated by summation of three independent data sets (STable 3). The strongest compression of −4.01 × 10^−5^ was obtained at Otsu hot spring by A1, located above the fault, while a total extension of 9.70 × 10^−6^ was obtained at Ajisai hot spring, the southwestern end of sampling sites (Fig. 1). There is an apparent positive correlation between the estimated strain changes and amounts of released helium at each site (Fig. 3). This is the first data set of quantitative linkage between seismological and geochemical observations, which would be explained by a physical mechanism such as release by rock fracturing. There was a laboratory study of helium degassing during rock fracturing subject to uniaxial compression^15^. When compressional loading of a rock exceeds half of the compressive strength to destroy, the volume of the rock undergoes an inelastic increase called “dilatancy” due to micro-cracking processes. Dilatancy may play an important role in triggering earthquakes^14^. When dilatancy of rock starts, new cracks are formed at grain boundaries and/or inside mineral grains. The surface area of the microfracture zones should increase in a rock by new cracks. Then helium in the vicinity of the newly exposed surface may be liberated from the crystalline lattice of mineral. If this is the case, there should be a positive correlation between the degassed helium and the degree of dilatancy. Based on the compression experiment of crustal rocks in vacuum, an equation governing empirical relationship between the residual fraction of helium in rock (R) and the dilatancy (ΔV/V) expressed in units of volumetric strain was proposed as follows:


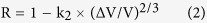


where k_2_ is a constant with the value of 25 ± 15 obtained by the experiment^15^. Following the equation, we may write the relation between the volumetric stain change and amount of helium (υ) released from aquifer rock as equation (1).

## Additional Information

**How to cite this article**: Sano, Y. *et al*. Groundwater helium anomaly reflects strain change during the 2016 Kumamoto earthquake in Southwest Japan. *Sci. Rep.* **6**, 37939; doi: 10.1038/srep37939 (2016).

**Publisher's note:** Springer Nature remains neutral with regard to jurisdictional claims in published maps and institutional affiliations.

## Supplementary Material

Supplementary Information

## References

[b1] Holland H. D. The Chemical Evolution of The Atmosphere and Oceans, 598 pp. (Princeton Univ. Press, 1984).

[b2] Self S., Widdowson M., Thordarson T. & Jay A. E. Volatile fluxes during flood basalt eruptions and potential effects on the global environment: A Deccan perspective. Earth Planet. Sci. Lett. 248, 518–522 (2006).

[b3] Black B. A., Elkins-Tanton L. T., Rowe M. C. & Peate I. U. Magnitude and consequences of volatile release from the Siberian Traps. Earth Planet. Sci. Lett. 317–318, 363–373 (2012).

[b4] Marty B. & Tolstikhin I. N. CO_2_ fluxes from mid-ocean ridges, arcs and plumes. Chem. Geol. 145, 233–248 (1998).

[b5] Wallace P. J. Volatiles in subduction zone magmas: concentrations and fluxes based on melt inclusion and volcanic gas data. J. Volc. Geotherm. Res. 140, 217–240 (2005).

[b6] Irwin W. P. & Barnes I. Tectonic Relations of Carbon Dioxide Discharges and Earthquakes. J. Geophys. Res. 85, 3115–3121 (1980).

[b7] Sano Y., Takahata N., Igarashi G., Koizumi N. & Sturchio N. C. Helium degassing related to the Kobe earthquake. Chem. Geol. 150, 171–179 (1998).

[b8] Lee H. . G. Massive and prolonged deep carbon emissions associated with continental rifting. Nature Geoscience 9, 145–149 (2016).

[b9] Roeloff E. A. Hydrologic Precursors to Earthquakes: A Review. PAGEOPH 126, 177–209 (1988).

[b10] King C.-Y. Gas Geochemistry Applied to Earthquake Prediction’ An Overview. J. Geophys. Res. 91, 12269–12281 (1986).

[b11] Ulomov V. I. & Mavashev B. Z. Forerunners of the Tashkent earthquake, in The Tashkent Earthquake of 26 April 1966. Akad. Nauk Uzb. SSR 188–192 (1971).

[b12] Igarashi G. . Groundwater radon anomaly before the Kobe earthquake in Japan. Science 269, 60–61 (1995). 17787704 10.1126/science.269.5220.60

[b13] Sugisaki R. & Sugiura T. Geochemical Indicator of Tectonic Stress Resulting in an Earthquake in Central Japan, 1984. Science 229, 1261–1262 (1985). 17770815 10.1126/science.229.4719.1261

[b14] Scholz C. H., Sykes L. R. & Aggarwal Y. P. Earthquake Prediction: A Physical Basis. Science 181, 803–810 (1973). 17816227 10.1126/science.181.4102.803

[b15] Honda M., Kurita K., Hamano Y. & Ozima M. Experimental studies of He and Ar degassing during rock fracturing. Earth Planet. Sci. Lett. 59, 429–436 (1982).

[b16] Koike K. . Controls on radon emission from granite as evidenced by compression testing to failure. Geophys. J. Int. 203, 428–436 (2015).

[b17] Kamaya N. . Overview of The 2016 Kumamoto Earthquake. JpGU Abst MIS34-P01 (2016).

[b18] Horiguchi K. & Matsuda J. Geographical distribution of ^3^He/^4^He ratios in north Kyushu, Japan: Geophysical implications for the occurrence of mantle-derived fluids at deep crustal levels. Chem. Geol. 340, 13–20 (2013).

[b19] Sano Y., Marty B. & Burnard P. Noble gases in the atmosphere. In The Noble Gases as Geochemical Tracers. Advances in Isotope Geochemistry (ed. Burnard P.) 17–31 (Springer-Verlag 2013).

[b20] Ozima M. & Podosek F. A. Noble Gas Geochemistry, 367 pp. (Cambridge Univ. Press, 1983).

[b21] Kennedy B. M. . Mantle Fluids in the San Andreas Fault System, California. Science 278, 1278–1281 (1997).

[b22] Dogan T. . Adjacent releases of mantle helium and soil CO_2_ from active faults: Observations from the Marmara region of the North Anatolian Fault zone, Turkey. Geochem. Geophys. Geosyst. 10, Q11009 (2009).

[b23] Klemperer S. L. . Mantle fluids in the Karakoram fault: Helium isotope evidence. Earth Planet. Sci. Lett. 366, 59–70 (2013).

[b24] Sano Y. & Wakita H. Geographical distribution of ^3^He/^4^He ratios in Japan: Implications for arc tectonics and incipient magmatism. J. Geophys. Res. 90, 8729–8741 (1985).

[b25] Rison W. & Craig H. Helium isotopes and mantle volatiles in Loihi Seamount and Hawaiian Island basalts and xenoliths. Earth Planet. Sci. Lett. 66, 407–426 (1983).

[b26] Sano Y. . Ten-year helium anomaly prior to the 2014 Mt Ontake eruption. Sci. Rep. 5, 13069, 10.1038/srep13069 (2015). 26286468 PMC4541341

[b27] Shibasaki T. Groundwater Basin and Groundwater Flow System. In Fluid Dynamics in a Deep Sedimentary Basin (Tokai Univ. Press) pp. 109–135 (1981).

[b28] Yurai H. . Crustal deformation of the 2016 Kumamoto Earthquake. JpGU Abst MIS34-03 (2016).

[b29] *GSI, Hypocenter Fault model of the 2016 Kumamoto Earthquake*. http://www.gsi.go.jp/common/000140781.pdf (2016).

[b30] Shimizu H. . Urgent joint seismic observation of the 2016 Kumamoto earthquake - Seismic activities and their background -. JpGU Abst MIS34-02 (2016).

[b31] Kumahara Y. . Distribution of surface rupture associated the 2016 Kumamoto earthquake and its significance. JpGU Abst MIS34-05 (2016).

[b32] Aoi S. . Strong motion and source processes of the 2016 Kumamoto earthquake sequence. JpGU Abst MIS34-06 (2016).

[b33] *Fire and Disaster Management Agency*, “*Disaster information*” http://www.fdma.go.jp/bn/2016/detail/960.html (2016).

[b34] Sano Y. & Wakita H. Precise Measurement of Helium Isotopes in Terrestrial Gases Bull. Chem. Soc. Japan 61, 1153–1157 (1988).

[b35] Okada Y. Internal deformation due to shear and tensile faults in a half-space. Bull. Seis. Soc. Am. 82, 1018–1040 (1992).

