# Supplementary material for: Groundwater helium anomaly reflects strain change during the 2016 Kumamoto earthquake in Southwest Japan
Source: Sci Rep. 2016 Nov 29;6:37939. doi: 10.1038/srep37939 (PMC5126567; doi:10.1038/srep37939)
Supplement: Supplementary Information [file srep37939-s1.pdf]

Supplementary Information

Groundwater helium anomaly reflects strain change during the 2016 Kumamoto earthquake in Southwest Japan

Yuji Sano[1*], Naoto Takahata[1], Takanori Kagoshima[1], Tomo Shibata[2], Testuji Onoue[3], Dapeng Zhao[4]

1. Atmosphere and Ocean Research Institute, The University of Tokyo, Kashiwa, Chiba 277-8564, Japan.
2. Institute for Geothermal Sciences, Kyoto University, Beppu, Oita 874-0903, Japan.
3. Department of Earth and Environmental Sciences, Kumamoto University, Kurokami, Kumamoto 860-8555, Japan.
4. Department of Geophysics, Tohoku University, Sendai, Miyagi 980-8578, Japan.

*Correspondence and requests for materials should be addressed to Y.S. (e-mail: ysano@aori.u-tokyo.ac.jp)

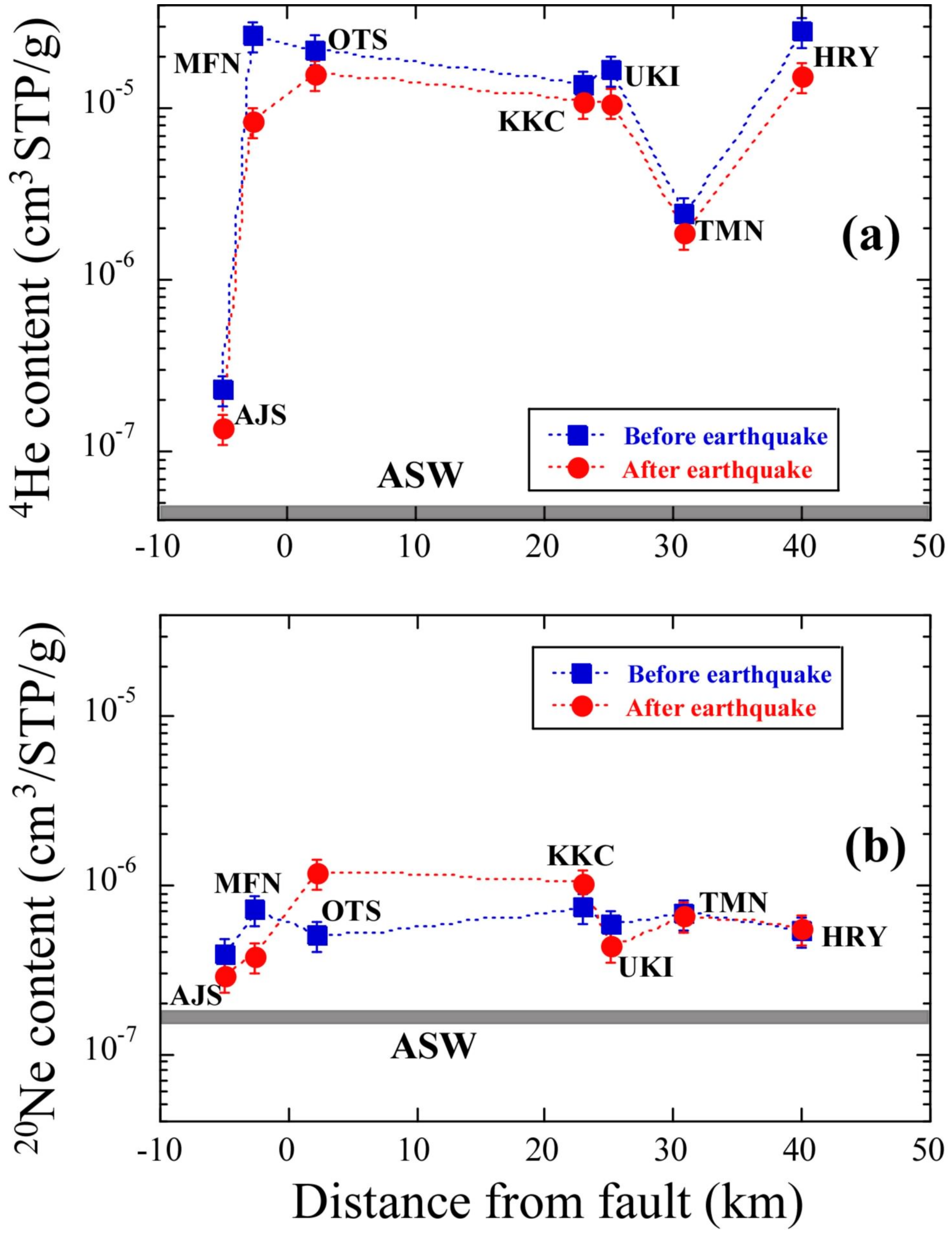

Supplementary Figure 1. (a) Relationship between the distance from the fault to the sampling site and helium content, and (b) that between the distance and neon content. ASW denotes the value of air saturated water.

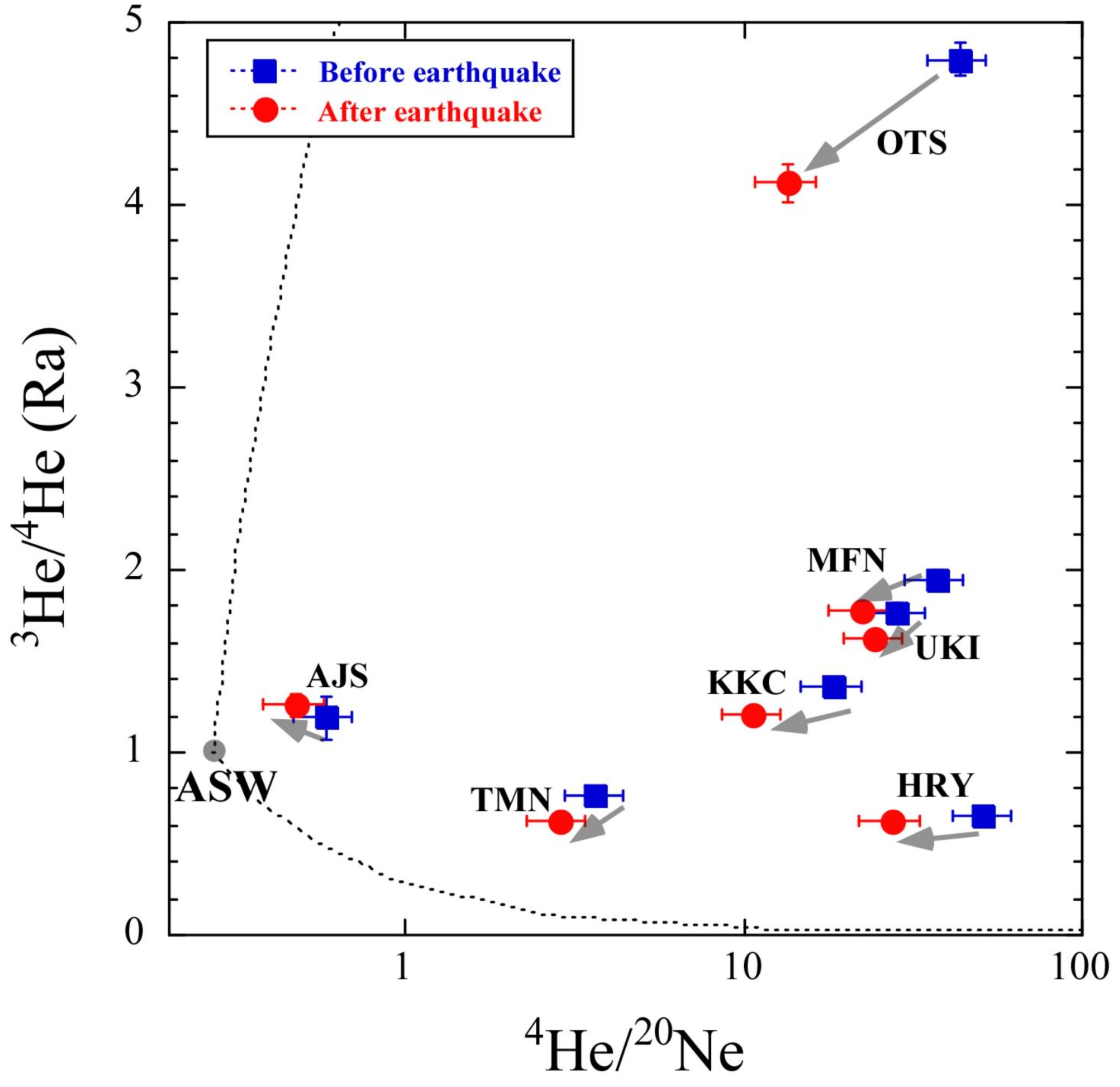

Supplementary Figure 2. Relationship between the $^4He/^{20}Ne$ and $^3He/^4He$ ratios of deep groundwater before and after the 2016 Kumamoto earthquake.

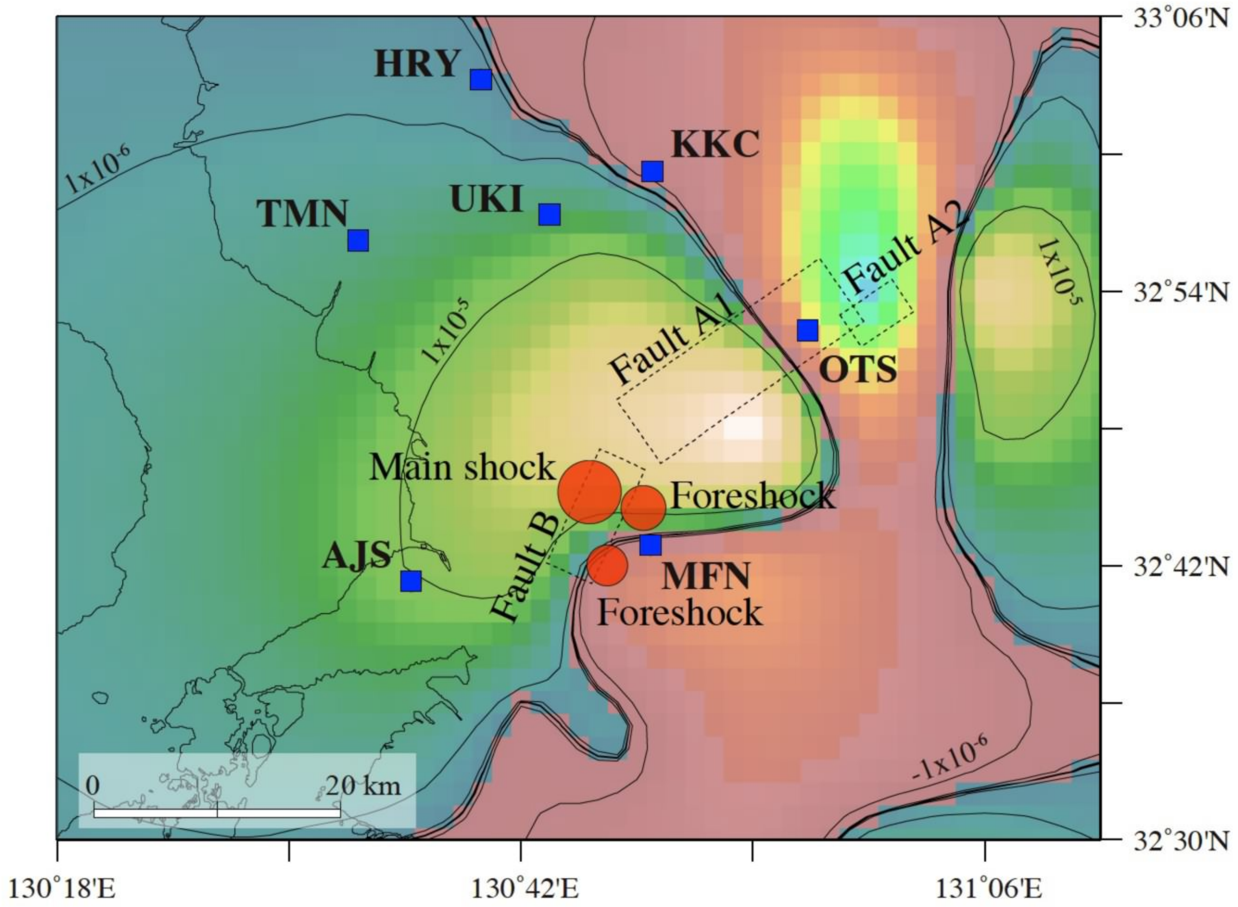

Supplementary Figure 3. Calculated volumetric strain changes in the Kumamoto region, Southwest Japan, which are estimated from three fault movements, A1, A2 and B (squares enclosed in dotted lines). Color contours show the strain changes; blue and red denote tensional and compressional changes, respectively. The blue squares denote the sampling sites. The base map was drawn by software package GMT32 (P. Wessel, W.H.F. Smith, New, improved version of the Generic Mapping Tools released. EOS Trans. AGU 79, 579, 1998).

STable 1. Sampling site, date, depth, $^4He/^{20}Ne$ and $^3He/^4He$ ratios of deep groundwater samples in Kumamoto region, SW Japan.

Code	Sampling site	Sampling date	Location		Depth	Distance	^4He content	$^4He/^{20}Ne$	$^3He/^4He$*	corrected $^3He/^4He$*
			Longitude (N)	Latitude (E)	(m)	(km)	(cm^3STP/g)		(Ra)	(Ra)
AJS	Ajisai	2016.4.28	32°41'17"	130°36'17"	900	-5.0	1.37E-07	0.474	1.260 ± 0.060	1.604 ± 0.111
MFN	Mifune	2016.4.29	32°42'52"	130°48'41"	500	-2.7	8.36E-06	22.1	1.780 ± 0.040	1.790 ± 0.040
OTS	Otsu	2016.4.28	32°52'16"	130°56'48"	1000	2.2	1.60E-05	13.5	4.120 ± 0.100	4.184 ± 0.102
KKC	Kikuchi	2016.4.28	32°59'12"	130°48'47"	300	23.0	1.10E-05	10.7	1.210 ± 0.020	1.215 ± 0.020
UKI	Ueki	2016.4.28	32°57'20"	130°43'28"	280	25.1	1.08E-05	24.5	1.620 ± 0.040	1.627 ± 0.040
TMN	Tamana	2016.4.28	32°56'13"	130°33'31"	400	30.8	1.88E-06	2.85	0.623 ± 0.020	0.584 ± 0.019
HRY	Hirayama	2016.4.28	33°03'12"	130°39'54"	1300	40.0	1.53E-05	27.6	0.630 ± 0.020	0.626 ± 0.020

*: Error is two sigma.

Distnce is calculated from the Futagawa-Hinagu fault.

STable 2. Helium contents and isotopic ratios of rock samples possibly composed in aquifer system in the Kumamoto region.

No.	Code	Sampling site	Rock name	Longitude (N)	Latitude (E)	He content (cm^3STP/g)	$^3He/^4He$ (Ra)	
1	MNT-1	Mt. Manotani	Pelitic schist	32˚41'07.2"	130˚53'40.8"	1.2E-06	0.07	± 0.13
2	MNT-2	Mt. Manotani	Mafic schist	32˚41'07.2"	130˚53'40.8"	6.9E-07	<0.01	
3	HGO-1	Kosa	Biotite gneiss	32˚38'34.3"	130˚49'50.9"	2.8E-06	0.25	± 0.05
4	KNH-1	Konoha	Pelitic schist	32˚55'29.8"	130˚39'46.6"	1.6E-06	0.16	± 0.08
5	SJN-1	Shimojin	Psammitic schist	32˚47'43.5"	130˚51'23.6"	2.8E-06	0.06	± 0.05
6	SJN-2	Shimojin	Mafic schist	32˚47'43.5"	130˚51'23.6"	2.4E-06	0.56	± 0.16
		Error weighted mean				1.9E-06	0.09	± 0.02

Error is two sigma.

STable 3. Corrected $^3He/^4He$ ratios of before and after the M7.3 earthquake, original helium abundance, additional helium and rock degassing helium together with strain change.

Code	Sampling site	Corrected $^3He/^4He$ (Ra) Before Eq.*	After Eq.	Original He* (cm^3STP/g)	Additional He (cm^3STP/g)	Rock degassing He (cm^3STP/g)	Strain change (ΔV/V) A1	A2	B	total
AJS	Ajisai#	1.424	1.604	2.32E-07	–	–	2.87E-06	1.16E-08	6.82E-06	9.70E-06
MFN	Mifune	1.959	1.790	2.66E-05	2.64E-06 ± 1.03E-06	1.05E-07 ± 4.12E-08	-1.56E-05	-7.79E-07	-6.84E-07	1.71E-05
OTS	Otsu	4.823	4.184	2.22E-05	3.46E-06 ± 6.73E-07	1.37E-07 ± 2.76E-08	-4.01E-05	1.02E-05	2.22E-06	5.25E-05
KKC	Kikuchi	1.366	1.215	1.37E-05	1.83E-06 ± 5.18E-07	7.25E-08 ± 2.09E-08	-2.04E-06	1.70E-06	-1.95E-06	5.69E-06
UKI	Ueki	1.775	1.627	1.68E-05	1.61E-06 ± 7.04E-07	6.40E-08 ± 2.81E-08	6.61E-06	1.09E-06	-3.72E-06	1.14E-05
TMN	Tamana	0.747	0.584	2.49E-06	8.16E-07 ± 1.74E-07	3.24E-08 ± 7.10E-09	4.40E-06	3.27E-07	-1.73E-06	6.46E-06
HRY	Hirayama	0.645	0.626	2.80E-05	9.67E-07 ± 1.43E-06	3.84E-08 ± 5.69E-08	4.46E-07	3.87E-07	-1.47E-06	2.30E-06

*: Data are refferred from Horiguchi and Matsuda (18).

#: Correction of helium isotopes is large due to low $^4He/^{20}Ne$ ratio. Data are not used in following discussion.

Error is two sigma.
